# The Relationship Between the Spatial Occurrence of *Leptospira* Exposed Animals and the Characteristics of the Peridomiciles They Inhabit in a Locality of Southeastern Mexico

**DOI:** 10.3390/pathogens13121037

**Published:** 2024-11-24

**Authors:** Alejandro Suárez-Galaz, Enrique Reyes-Novelo, Anabel Cruz-Romero, Rodrigo Ramos-Vázquez, Jesús Alonso Panti-May, Hugo Ruiz-Piña, Sokani Sánchez-Montes, Marco Torres-Castro

**Affiliations:** 1 Laboratorio de Zoonosis y otras Enfermedades Transmitidas por Vector, Centro de Investigaciones Regionales “Dr. Hideyo Noguchi”, Universidad Autónoma de Yucatán, Mérida 97000, Yucatán, Mexico; alexrsg97@gmail.com (A.S.-G.); enrique.reyes@correo.uady.mx (E.R.-N.); alonso.panti@correo.uady.mx (J.A.P.-M.); rpina@correo.uady.mx (H.R.-P.); 2 Laboratorio de Enfermedades Infecciosas, Facultad de Medicina Veterinaria y Zootecnia, Universidad Veracruzana, Carretera Veracruz, Xalapa 91697, Veracruz, Mexico; anabelcruzromero84@gmail.com (A.C.-R.); rodrigo_1393@live.com (R.R.-V.); 3 Facultad de Ciencias Biológicas y Agropecuarias, Universidad Veracruzana, Región Poza Rica-Tuxpan, Tuxpan de Rodríguez Cano 92870, Veracruz, Mexico; sok10108@gmail.com; 4 Centro de Medicina Tropical, Unidad de Investigación en Medicina Experimental, Facultad de Medicina, Universidad Nacional Autónoma de México, Ciudad de México 06720, Mexico

**Keywords:** leptospirosis, rodents, dogs, opossums, epidemiology, zoonotic diseases

## Abstract

The occurrence of *Leptospira* in mammals is an indicator for assessing potential health risks, particularly in tropical regions. Understanding their hosts’ habitat characteristics and spatial occurrence is essential to surveil them. This study aimed to determine the characteristics of the peridomiciles associated with the spatial occurrence of *Leptospira* hosts. We inventoried the characteristics of the peridomiciles. Blood serum was gathered from dogs, opossums, and rodents for a microagglutination essay to detect exposure to several serogroups of *Leptospira*. A generalized linear model with binomial distribution helped estimate risk ratios and 95% confidence intervals between a seropositive animal’s occurrence and the peridomiciles’ characteristics. For estimates with the occurrence of one or more seropositive animals, a multinomial model was fitted. The seroprevalence in rodents was 81.8%; in opossums 31.2%; and 56.5% for dogs. The most frequent serogroup in dogs and rodents was Canicola. In opossums, the seroreaction was against Tarassovi, Ballum, Sejroe, and Cynopteri. The results showed that the peridomicile area (m^2^), the geographic polygon, and the accumulation of plastic containers (PET) were characteristics related to the occurrence of seropositive animals. The results revealed that the peridomicile’s characteristics and spatial distribution in the locality help explain the occurrence of *Leptospira* hosts.

## 1. Introduction

Leptospirosis is a neglected zoonotic disease caused by pathogenic spirochetes of the genus *Leptospira* [1]. This disease is highly prevalent in tropical and subtropical areas of the world, where communities of rodent reservoirs are favored by sanitary deficiencies and the availability of food and shelter sources, causing a suitable environment to maintain the transmission [2,3].

Currently, the *Leptospira* genus includes 70 valid species divided phylogenetically into two groups: pathogenic (P) and saprophyte (S) [4,5]. The pathogenic species had 32 serogroups with approximately 320 serovars based on their homology, reaction, and antigenic structures [6]. These pathogenic species are responsible for generating approximately 1.03 million cases of human Leptospirosis and sixty thousand deaths annually in the world, with a high burden in American countries [7]. A recent study estimated Leptospirosis prevalences of 28% in North America, 31% in Central America, and 26% in South America [8].

In Mexico, during 2013–2019, the highest incidence in the southeastern states was associated with biotic and abiotic factors such as natural reservoir populations (rodents) and climatic conditions, which allow bacteria to survive in humid environments where susceptible hosts, including humans, come into contact [9]. Specifically, in Yucatan, during 2020–2023, 54 cases of human Leptospirosis were reported, representing 6.3% of the accumulated cases for Mexico, positioning it as the third state with the highest number of reports [10].

The state of Yucatan has a historical record of research on *Leptospira* in anthropized and sylvatic areas gathering knowledge on the frequency in the human population [11] and the diversity of its animal hosts, which includes wild mammals such as rodents [12,13,14,15], bats [15,16,17], opossums [11,18], shrews [19], pets and domestic fauna such as cats, dogs [20,21,22], cows, and pigs [11,23], respectively.

Epidemiological studies in tropical and subtropical endemic regions have identified that in areas with human settlements, the presence of synanthropic fauna (e.g., rodents and opossums), the characteristics of the vegetation cover in the peridomicile or adjacent areas, the proximity to public sewage services, and the unintentional storage of wastewater are factors associated with the transmission of *Leptospira* [2,24,25]. In this context, in southeastern Mexico, the peridomicile is characterized by structures made and maintained around the house for storing belongings, raising animals, and other economic activities relevant to the group or family of people who manage it [26,27]. This space offers resources such as shelter and food to wild (e.g., rodents), synanthropic, and domestic (e.g., dogs, cats, etc.) fauna temporarily or permanently occupying it. Such a fauna generally hosts endemic zoonotic pathogens, including *Leptospira* [13,14,28], implying a greater risk of exposure for people living in the house and their domestic and companion animals [29].

Given the circulation of *Leptospira* in several mammal species, identifying factors associated with their occurrence is essential to understanding its ecoepidemiology. In this regard, Suárez-Galaz et al. [15] determined that in sylvatic areas, the forest cover and the diversity of susceptible host species are factors involved in the occurrence of *Leptospira* hosts; however, in the case of human settlements in tropical areas or Mexico, these factors have not been studied. Our study aimed to determine the characteristics of the peridomiciles associated with the occurrence of animals exposed to pathogenic *Leptospira*, looking for a better understanding of its ecoepidemiology in the synanthropic context.

## 2. Materials and Methods

### 2.1. Site and Study Design

**Figure 1:**
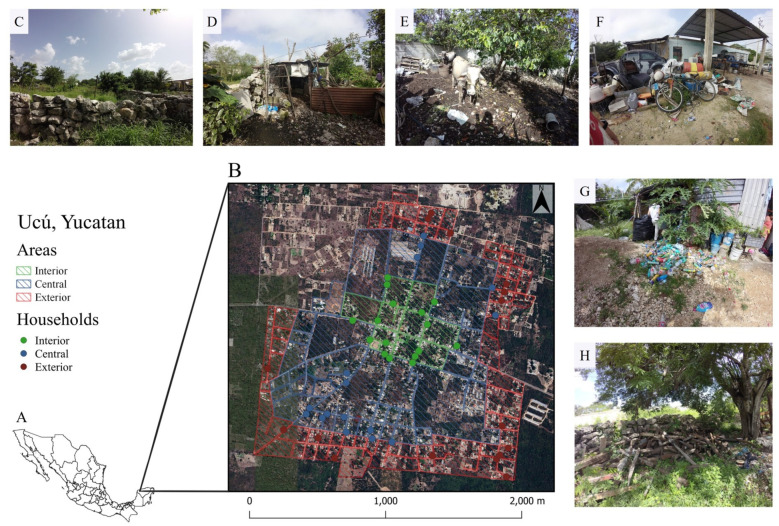
The polygon of Ucú, Yucatan, showing the geographic location and some peridomicile characteristics. (**A**): Map of Mexico, (**B**): Map of Ucú, (**C**): Stone wall, (**D**): Buildings for animal husbandry, (**E**): Production animals, (**F**): Accumulation of miscellaneous items, (**G**): Accumulation of PET, (**H**): Accumulation of wood.

The study site was the town of Ucú, Yucatan, Mexico (21°01′55′′ N, 89°44′47′′ W) (Figure 1A). The municipality has an area of 130.8 km^2^ and an altitude of 8 m above sea level. The average annual temperature is 26 °C, and yearly annual accumulated precipitation is 600 to 800 mm. The locality has 4049 inhabitants spread over 1139 homes [30,31].

The study was cross-sectional, with a sample size estimated to obtain the proportion of peridomiciles with at least one synanthropic animal using 95% confidence, 5% precision, and an expected 6% of homes with synanthropic animals, according to other similar studies in the region [12,14,32,33], and following the criteria of Thrusfield [34] with a final minimum number of peridomiciles included in the study of 48.

The peridomiciles included in the study were conditioned to the house owners agreeing to participate voluntarily by signing the informed consent form. The spatial distribution of the peridomiciles was classified by drawing two imaginary lines crossing the center of the locality’s polygon. Subsequently, three concentric circles were drawn to create areas (interior, central, and exterior) to balance the number of peridomiciles in each [35] (Figure 1B).

### 2.2. Sampling and Population Data

The capture of rodents and opossums in peridomiciles was during August and September 2021 by placing ten Sherman traps (7.5 cm × 23 cm × 9 cm, HB Sherman Traps Inc^®^, Tallahassee, FL, USA) baited with a mixture of oat flakes and artificial vanilla and a Tomahawk trap (Tomahawk Livetraps^®^, Hazelhurst, WI, USA) baited with fresh fruit, during two consecutive nights per peridomicile [36]. The capture effort for rodents was 960 h/trap and 96 h/trap for opossums. The captured animals were transported to the Regional Research Center (CIR in Spanish) “Dr. Hideyo Noguchi”—Autonomous University of Yucatan (UADY in Spanish) to obtain biological samples.

Rodents were desensitized and anesthetized with isoflurane (Piramal Enterprises Limited^®^, West Drayton, LDN, United Kingdom). A blood sample was collected intracardially using sterile ultra-fine syringes (BD Ultra-FineTM, Mexico City, MEX, Mexico). Subsequently, rodents were euthanized by an overdose of sodium pentobarbital (Aranda^®^, Queretaro, QRO, Mexico) [37]. In opossums, blood was collected using sterile syringes (21 G × 32 mm) (BD Plastipak^TM^, Mexico City, MEX, Mexico) by puncture of the caudal vein [36]. They were then released into forest patches close to the study site with non-permanent markings for identification in case of recapture. All blood samples were placed in 1.8 mL microcentrifuge tubes (Eppendorf^®^, Hamburg, Germany) without anticoagulant. These were left at room temperature (24 °C) for 20 min to retract the clots, and the serum was separated into new tubes and stored frozen (−20 °C) until use.

When the homes had dogs, a blood sample was taken from each during peridomicile visits with prior authorization from their custodians. Blood samples (up to 5 mL) were obtained by puncturing the cephalic or saphenous vein with sterile syringes (21 G × 32 mm) (BD Plastipak^TM^, Mexico City, MEX, Mexico). The blood (approximately half) was placed in tubes with (BD Vacutainer^®^ K2 EDTA, Franklin Lakes, NJ, USA) and without anticoagulant (BD Vacutainer Serum^®^, Franklin Lakes, NJ, USA) and kept in portable refrigerators for transport to the laboratory.

For all animals, data on sex (male or female) and age were recorded (rodents and opossums: adult or juvenile [33,38]; dogs: puppy < 1 year, adult > 1 year up to 6 years, geriatric > 6 years).

### 2.3. Microscopic Agglutination Test (MAT)

The MAT procedure was according to the World Organization for Animal Health [39]. Blood samples were centrifuged at 3500 rpm for 10 min to obtain sera that were challenged against antigens of the following strains: *L*. *interrogans*, serogroup Canicola, serovar Canicola, strain Hond utrecht IV; *L*. *interrogans*, serogroup Icterohaemorrhagiae; *L*. *interrogans*, serogroup Hardjo; *L*. *interrogans*, serogroup Pomona, serovar Pomona, strain Pomona; *L*. *interrogans*, serogroup Pyrogenes, serovar Pyrogenes, strain Salinem; *L*. *interrogans*, serogroup Bataviae, serovar Bataviae, strain Paidjan; *L*. *interrogans*, serogroup Australis, serovar Bratislava, strain Jez Bratislava; *L*. *borgpetersenii*, serogroup Tarassovi, serovar Tarassovi, strain Perepelitsin; *L*. *borgpetersenii*, serogroup Ballum, serovar Ballum, strain Mus 127; *L*. *borgpetersenii*, serogroup Sejroe, serovar Sejroe, strain M84; *L*. *kirschneri*, serogroup Grippotyphosa, serovar Grippotyphosa, strain CAL4; *L*. *kirschneri*, serogroup Cynoptery, serovar Cynoptery, strain 3522 C; and *L*. *noguchii*, serogroup Panama, serovar Panama, strain CZ 214 K.

Sera were “seropositive” if they showed 50% or more agglutination at initial dilutions ≥1:100 for rodents, ≥1:200 for opossums, and ≥1:400 for dogs [39,40]. A particular serogroup was determined when the sera had the highest titer reaction to a serogroup. Some sera reacted to more than one serogroup and were classified consequently.

### 2.4. Peridomicile Data Collection

The collection of data from peridomiciles was using a semi-structured questionnaire that contained characteristics related to the occurrence of wild (rodents) or/and synanthropic (rodents and opossums) fauna in peridomiciles based on variables regarded relevant in previous epidemiological studies carried out in southern Mexico and other American countries [24,25,41,42,43,44].

The characteristics recorded in the questionnaire were area of the town (interior, central, exterior, see Figure 1), geographic polygon (northeast, northwest, southeast, southwest), type of neighboring (houses, uninhabited properties, public areas [parks, markets]), peridomicile delimiter (block wall, stone wall [known in the region as “*albarrada*” (Figure 1C)], wall with other materials, without wall), peridomicile area (in m^2^), type of floor (dirt or dirt and concrete), main vegetation cover (trees, shrubs, and herbaceous), dominant vegetation (trees or herbaceous), number of trees, diversity of trees (number of species), buildings in the peridomicile (Figure 1D) (presence or absence of warehouse, barn, and buildings for animal husbandry), production animals (Figure 1E) (absence or presence of animals such as pigs, cows, poultry, among others), captive wildlife (presence or absence of wild animals), accumulation of miscellaneous items (Figure 1F) (presence or absence of polyethylene terephthalate (PET, Figure 1G) containers, cans, cardboard, pots, firewood, construction materials, cement blocks, wood (Figure 1H), construction clay, construction rubbish, gravel), and presence or absence of water containers (lids, plastic containers, glasses, cups, sinks, ponds, tubs or animal waterers).

### 2.5. Data Analysis

The overall and specific seroprevalence for rodents, opossums, and dogs with their confidence intervals (95% CI) were estimated by the Clooper-Pearson method with the Quantitative Parasitology^®^ package [45].

Bivariate analyses were performed with Fisher’s exact test to determine the association between the occurrence of a seropositive animal and the characteristics of the peridomiciles—those variables with a *p*-value ≤ 0.3 adjusted six generalized linear models with binomial distribution. The first model was with the peridomicile area. The second model was adjusted with the number of trees. The third model was adjusted with the diversity of trees. The fourth model was adjusted by adding the peridomicile area’s effect plus the diversity of trees. The fifth model consisted of a null model (intercept). The sixth model was adjusted with the variables peridomicile area and geographic polygon. The models were compared using the Akaike criterion (AIC) and a chi-squared analysis to compare the deviances and select the final model with the best fit. Subsequently, risk ratios (RR) and their 95% CI were estimated for statistically significant variables (*p* ≤ 0.05).

Since more than one host may be present in a peridomicile, the variables related to the occurrence of one or more *Leptospira*-seropositive animals were determined. For this, bivariate analyses were performed using Fisher’s exact test. Those with a *p*-value ≤ 0.3 were used to adjust a multinomial model using the AIC criterion for the final selection, and RR and 95% CI were estimated for statistically significant variables (*p* ≤ 0.05).

Statistical analyses were performed in the Rstudio programming environment (Posit Team, 2024) using the R v.4.4 language [46,47], employing the packages “car” [48], “ExpDEs” [49], “nortest” [50], “lsmeans” [51], “emmeans” [52], “phia” [53], “ggplot2” [54], “foreing” [47], “nnet” [55], “reshape2” [56], “nlme” [57], “MASS” [58], and “RcmdrMisc” [59].

## 3. Results

In total, 60 animals were captured: 36 rodents belonging to three species (*Mus musculus*, *Peromyscus yucatanicus*, and *Ototylomys phyllotis*) and 24 opossums (*Didelphis virginiana*). The dogs sampled in the peridomiciles were 66 (Table 1).

Overall, 70.8% (34/48) of the studied peridomiciles had animals (rodents or/and opossums) and 77.1% (37/48) had at least one sampled dog. Houses located in the exterior area of Ucú had more rodent captures and sampled dogs compared with the other areas. In contrast, opossums were more frequent in peridomiciles in the central area (Table 2).

Blood samples were obtained from 16/24 opossums, 33/36 captured rodents, and 62/66 sampled dogs. Therefore, 111 sera were processed for MAT diagnosis (Table 2). The overall seroprevalence was 60.4% (95% CI 50.6–69.5%). The seroprevalence for rodents was 81.8% (95% CI 64.5–93%), 31.2% (95% CI 11–58.7%) for opossums, and 56.5% (95% CI 43.3–69%) for dogs (Table 1).

The highest frequency of reactive sera for the evaluated serogroups was against Canicola, with 28.3% (19/67). In contrast, no reactive sera were observed against the Australis serogroup. The highest seroreaction in rodents and dogs was against the Canicola serogroup, with 26% (7/27) and 34.3% (12/35), respectively. In opossums, the highest seroreaction was against the Cynopteri serogroup with 40% (2/5) (Table 3).

The characteristics of the peridomiciles studied showed that 48% (23/48) were adjacent (type of neighboring) to uninhabited properties. Most peridomicile delimiters were stone walls and walls built with other materials, with 31.2% (15/48) in both cases.

Overall, 58.3% (28/48) of the peridomiciles had trees as the dominant form of vegetation. Constructions for animal husbandry (chicken coops, pigpens, corrals) occurred in 47.9% (23/48) of the peridomiciles. The items most frequently accumulated by the inhabitants were pots 89.5% (43/48), firewood 81.2% (39/48), and PET containers 79.2% (35/48). Finally, containers that accumulate water were in 93.7% (54/48) of the peridomiciles (Table 4).

**Figure 2:**
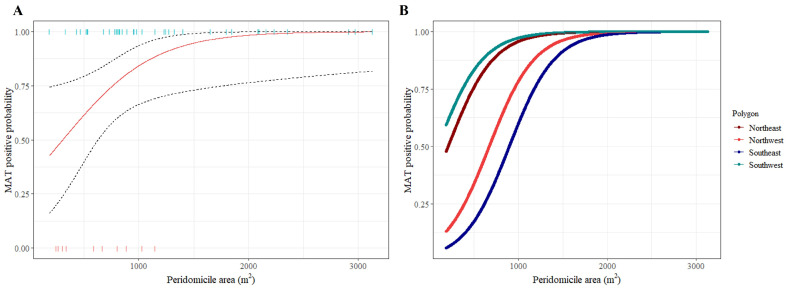
(**A**) Binomial generalized linear model fit estimating the probability of finding a seropositive animal according to the peridomicile area (m^2^). The dotted lines represent the 95% confidence interval of the estimate. (**B**) Binomial generalized linear model fit estimating the probability of finding a seropositive animal according to the peridomicile area (m^2^) adjusted to the geographic polygon of Ucú, Yucatan, Mexico.

Analyses using Fisher’s exact test showed a statistical association (*p* ≤ 0.3) between the occurrence of seropositive animals and the following variables: geographic polygon (*p* = 0.24), peridomicile delimiter (*p* = 0.12), presence of buildings for animal breeding (*p* = 0.29), presence of captive wildlife (*p* = 0.3), accumulation of PET (*p* = 0.1), and the peridomicile area (*p* = 0.03) (Table 4, Figure 2A).

The evaluation of the different generalized linear models showed that the best model was fitted with the predictors “peridomicile area” and the “geographic polygon” to estimate the probability of at least one seropositive animal occurring (Table 5). This model predicts that for each m^2^ increase in the peridomicile area, the risk of finding a seropositive animal increases 0.003 times (95% CI = 0.001–0.008) (Table 4; Figure 2A) and that in peridomiciles with an area greater than 1500 m^2^, the probability of finding seropositive animals is very high (Figure 2A).

Regarding the geographic polygon, the model shows that peridomiciles situated within the southeast polygon are less likely to host a *Leptospira*-seropositive animal (RR = 0.07, CI95 = 0.002–0.80) compared to peridomiciles situated in the other polygons (Table 4) and that for peridomiciles situated in the northeast and southwest polygons, the risk of finding seropositive animals is higher in those with areas smaller than 1500 m^2^ (Figure 2B).

Exploratory bivariate analyses with Fisher’s exact test (*p* ≤ 0.3) showed that the presence of one or more seropositive species in the peridomicile was associated with the peridomicile area (*p* = 0.007), the peridomicile delimiter (*p* = 0.3), the type of floor (*p* = 0.28), the tree cover (*p* = 0.24), the herbaceous cover (*p* = 0.05), the dominant vegetation (*p* = 0.20), the presence of a warehouse (*p* = 0.29), and the accumulation of PET (*p* = 0.03), cans (*p* = 0.005) and pots (*p* = 0.15) (Table 6). The adjustment of these variables to the multinomial model (Table 6) showed that peridomiciles with an accumulation of PET have a greater chance of having a seropositive animal species (RR = 8.52 C95% = 1.38–52.56); however, the intercept of this model was not significant.

## 4. Discussion

Our study revealed that the peridomicile’s characteristics and spatial distribution in the locality help to explain the occurrence of *Leptospira*-exposed animals. The expansion of several anthropogenic activities has led to an increasing interaction between pathogens, people, reservoirs, and susceptible hosts, leading to the emergence or reemergence of zoonoses like Leptospirosis [60]. One of the anthropogenic spaces in which these interactions occur is the peridomicile area, becoming a scenario for the occurrence of enzootic and zoonotic transmission of pathogens carried by wild or synanthropic fauna and transmitted to susceptible hosts such as pets, domestic animals, and people living in dwellings [27,29].

Some of this fauna (rodents, opossums, and dogs) has been reported as a host of *Leptospira* in southeastern Mexico [12,14]. In addition, some studies have identified characteristics of the peridomicile that favor its occurrence [29]. However, many specific factors associated with the occurrence and circulation of animals carrying each pathogen, including *Leptospira*, are unknown. The seroprevalence of *Leptospira* in the tested animals was 60%. This finding is relevant to people because synanthropic fauna (rodents and opossums) and dogs exposed to these bacteria in endemic regions are highly associated with an increased risk of transmission to the house’s inhabitants [60].

Biotic factors (e.g., communities of natural reservoirs and susceptible hosts) and abiotic factors (e.g., climatic conditions such as high rainfall, temperature, and relative humidity) participate in maintaining the transmission of *Leptospira* between reservoirs and hosts [7,60,61]. Eighty-two percent of the tested rodents of Ucú were reactive to at least one *Leptospira* serogroup. This frequency is higher than in other studies conducted with rodents of Mexico, such as the report in *Rattus rattus* (synanthropic) (15%) of Yucatan [11] and in wild rodents (50%) captured in Tamaulipas, northeast of Mexico [62]. It also shows that seroprevalence could be highly variable between endemic regions. The presence of antibodies against *Leptospira* in rodents is explained by their interaction with pathogenic *Leptospira* from an early age through direct contact with the infected mother or with the burrow contaminated with urine [12,63].

The seroprevalence found in *D*. *virginiana* (31.2%) is higher than that reported for this mammal in Yucatan by Vado-Solis et al. [11] (5%) and Ruiz-Piña et al. [18] (4.9%). Likewise, it is higher compared to the records for *Didelphis albiventris* (2%, 3.4%) and *Didelphis aurita* (3.5%) from Brazil [64,65]. The production of antibodies against *Leptospira* in *Didelphis* marsupials starts when the individual encounters the urine excreted by other infected hosts or reservoirs in the area they share [66]; such contact can implicate *Didelphis* in the transmission as renal carriers of *Leptospira* pathogenic species although they are considered accidental hosts of these bacteria [67]. In this regard, the evidence obtained in this survey suggests that future studies should consider obtaining and analyzing urine or kidney tissue to determine whether *Didelphis* opossums can eliminate bacteria through urine, as has been observed in other studies [68,69]. This would provide a deeper understanding of their role in the ecology and epidemiology of *Leptospira*.

An interesting finding was that none of the opossums included in the serological test showed antibodies against *L*. *interrogans* serogroups (but see [11,18]), suggesting recurring contact with areas contaminated by reservoirs or hosts of *L*. *kirschneri* and *L*. *borgpetersenii* such as cattle, sheep, and pigs. Our work provides evidence suggesting that opossums may increase their interactions with production animals. This underscores the need to assess the potential impacts of the production of animals on marsupial communities in Mexico. Understanding these dynamics is crucial for wildlife health. Future studies should focus on pathogen spillover risks and the role of these marsupials in the transmission of diseases in the tropical regions of Mexico. Additionally, the omnivorous habits of opossums may induce them to have closer contact with sites with production-animal husbandry when searching for food or shelter [70].

The seroprevalence found in dogs (56%) of Ucú was higher than those previously reported in dogs of Yucatan by Vado-Solis et al. [11] (19%), Jimenez-Coello et al. [20] (35%), Ortega-Pacheco et al. [71] (34%), Cardenas-Marrufo et al. [72] (36%), and Ortega-Pacheco et al. [21] (45%), but less than Cruz-Romero et al. [73] (100%) in Mexico City; and this is also true for studies on dogs from states like Campeche (21.3%) [74] and Chiapas (4.9%) [75].

*Leptospira* transmission in dogs can be incidental because of direct contact with reservoirs and other hosts or contaminated environments [76,77,78], but dogs could have a different role in the epidemiology of *Leptospira*, because, in some endemic areas, they are “maintenance hosts” of *L*. *interrogans* serovar Canicola, keeping the zoonotic transmission cycle of this bacteria active, although there are also reports of cases of severe infections with this serovar and zoonotic transmission to their guardians [79,80].

The associations between the occurrence of animals with exposure evidence against *Leptospira* and some characteristics of peridomiciles, such as the peridomicile area, the geographic polygon, the peridomicile delimiter (Figure 1C), the presence of buildings for animal husbandry (Figure 1D), the presence of captive wildlife, and the accumulation of PET (Figure 1G), represent a first approximation to the complex ecology of the transmission of *Leptospira* in tropical areas.

Although some of these characteristics have been included in studies with similar objectives in other parts of the world [2,81], stone walls (Figure 1C) and other materials and buildings for animal husbandry (Figure 1D) are shared by most peridomiciles in localities in southeastern Mexico and are associated with the presence of synanthropic animals (rodents and opossums) [70,82,83].

Conversely, accumulating belongings (Figure 1F), pots, and inorganic waste, such as PET (Figure 1G), are also recurrent items associated with the occurrence of synanthropic fauna (rodents and opossums) in the peridomiciles because they represent shelter [44,70,83], and our results also found a direct relationship with the occurrence of *Leptospira* hosts. This finding is relevant because many families accumulate these types of articles to sell them to recycling companies and contribute to the family economy [44], pointing to the need to improve the handling and storage practices of these items to reduce the transmission potential of *Leptospira*.

The presence of captive wildlife was another characteristic associated with the occurrence and circulation of *Leptospira* hosts. In several localities of Yucatan, this fauna (mainly mammals) is expected to be observed in homes due to traditional cultural relevance [84,85]. The association with the presence of these animals in peridomiciles reveals the potential carrying of *Leptospira*, favoring its permanence in the human environment, as has been observed in other tropical regions of America [64,86,87]. Future studies must include all animal species in the peridomicile to corroborate and quantify their interaction with pathogenic *Leptospira*.

The results show two relevant aspects of the epidemiology of *Leptospira* at the study site. The first is the spatial occurrence at the locality level, and the second is at the peridomicile level. The data analysis showed that, at the locality level, peridomiciles set in the exterior area have more risk of sheltering more than one species of *Leptospira*-seropositive hosts, particularly the northeast and southwest polygons. This result seems to be supported by the abundance of rodents and dogs observed in this area and, consequently, a higher frequency of seropositive animals, as well as by the closeness to zones with surrounding secondary vegetation and agricultural areas since these habitats favor the interaction between the species, compared to peridomiciles in the innermost areas of the locality [88,89,90].

At the peridomicile level, the results show two relevant aspects for *Leptospira* hosts: the peridomicile area and the accumulation of PET. Regarding the peridomicile area, the probability of presenting at least one seropositive animal increases by 0.3% for each m^2^ that the area increases. Likewise, the binomial model allows us to predict that when peridomiciles are larger than 1500 m^2^, the probability of finding a seropositive animal is remarkably high. The size of the area is important due to the availability of resources and permanent or temporary refuge sites for these animals [44,70,83].

Peridomiciles with an accumulation of PET (Figure 1G) are more likely to have at least one seropositive animal. Although this association is not conclusive, it helps to explain how these materials accumulate in copious quantities and, for a sufficient time, become a potential refuge for synanthropic fauna, particularly for rodents [44,91]. In addition, they can regularly accumulate tiny amounts of rainwater to maintain infective *Leptospira* in the environment. The practice of accumulating this type of container is common among families living in poverty or extreme poverty since their accumulation and sale in volume represent an income that contributes to the family economy [92].

Finally, this study shows evidence that multiple hosts of pathogenic *Leptospira* occur and circulate in peridomiciles. These hosts follow a spatial distribution at the local level, limited by the peridomicile factors. The results show the need to develop measures to prevent the potential transmission of *Leptospira* to inhabitants, pets, and domestic animals.

## Figures and Tables

**Table 1:** Population data and seroprevalence against *Leptospira* of animals studied in peridomiciles of Ucú, Yucatan.

Species	n	Sex		Age (%)	MAT	Seroprevalence (IC 95%)
		♂ (%)	♀ (%)			
*Mus musculus* (synanthropic)	32	22 (68.8)	10 (31.2)	J: 5 (15.6) A: 27 (84.4)	23/29	81.8% (64.5–93) *
*Peromyscus yucatanicus* (wild)	3	2 (66.7)	1 (33.3)	A: 3 (100)	3/3	
*Ototylomys phyllotis* (wild)	1		1 (100)	A: 1 (100)	1/1	
*Didelphis virginiana* (synanthropic)	24	11 (45.8)	13 (54.2)	J: 8 (33.4) A: 16 (66.6)	5/16	31.2% (11–58.7)
*Canis lupus familiaris*	66	39 (59.1)	27 (40.9)	C: 12 (18.2) A: 47 (71.2) G: 7 (10.6)	35/62	56.5% (43.3–69)
Total	118				67/111	60.4% (50.6–69.5)

MAT: Positive sera/Analyzed sera * Seroprevalence by the rodent community.

**Table 2:** Number of animals captured and studied in peridomiciles of Ucú, Yucatan.

Species	Individuals/Analyzed Sera			
	Areas			
	Interior	Central	Exterior	Total
*Mus musculus* (synanthropic)	10/10	5/3	17/16	32/29
*Peromyscus yucatanicus* (wild)	--	1/1	2/2	3/3
*Ototylomys phyllotis* (synanthropic)	--	--	1/1	1/1
Rodent total	10/10	6/4	20/19	36/33
*Didelphis virginiana* (synanthropic)	11/7	8/5	5/4	24/16
*Canis lupus familiaris*	19/18	21/20	26/24	66/62
Total	40/35	35/29	51/47	126/111

**Table 3:** Frequency of reactive hosts’ sera against *Leptospira* serogroups tested by microscopic agglutination from Ucú, Yucatan, Mexico.

Species	Serogroup	Hosts			Total n (%)
		Rodents n (%) ≥ 1:100	Opossums n (%) ≥ 1:200	Dogs n (%) ≥ 1:400	
*L. noguchii*	Panama	2 (7.4)	--	--	2 (3)
*L. borgpetersenii*	Tarassovi	--	1 (20)	3 (8.6)	4 (6)
	Ballum	--	1 (20)	--	1 (1.5)
	Sejroe	6 (22.2)	1 (20)	8 (23)	15 (22.4)
*L*. *interrogans*	Pyrogenes	--	--	5 (14.2)	5 (7.5)
	Bataviae	3 (11.1)	--	1 (2.8)	4 (6)
	Canicola	7 (26)	--	12 (34.2)	19 (28.3)
	Australis	--	--	--	--
	Icterohaemorrhagiae	--	--	2 (5.8)	2 (3)
	Hardjo	1 (3.7)	--	--	1 (1.5)
	Pomona	4 (14.8)	--	1 (2.8)	5 (7.4)
*L*. *kirschneri*	Grippotyphosa	3 (11.1)	--	2 (5.8)	5 (7.4)
	Cynopteri	--	2 (40)	--	2 (3)
	More than one serogroup	1 (3.7)	--	1 (2.8)	2 (3)
Total		27	5	35	67

**Table 4:** Bivariate analysis (Fisher’s exact test) and generalized linear model (binomial) to evaluate the relationship between the characteristics of the peridomiciles with the presence of animals seropositive against *Leptospira* (n = 48 peridomiciles).

Peridomicile Characteristics	Number of Peridomiciles (%)	Bivariate Analysis (*p* ≤ 0.3)	Multivariate Analysis (*p* ≤ 0.05)	RR (CI 95%)
*Presence of seropositive animal*	38 (79.2)			
*Locality’s area*		1		
Interior	16 (33.3)			
Central	16 (33.3)			
Exterior	16 (33.3)			
*Geographic polygon*		0.24 *		
Northeast	10 (20.8)		Reference	
Northwest	9 (18.8)		0.24	0.16 (0.005–2.94)
Southeast	12 (25)		0.06	0.07 (0.002–0.80)
Southwest	17 (35.4)		0.74	1.59 (0.06–25.98)
*Type of neighboring*		1		
Houses	11 (22.9)			
Inhabited properties	23 (48)			
Public areas	14 (29.1)			
*Peridomicile delimiter*		0.12 *		
Block wall	4 (8.4)			
Stone wall	15 (31.2)			
Wall built with diverse materials	15 (31.2)			
Without delimiter	14 (29.2)			
*Peridomicile area* (m^2^)	1129.02 ± 741.48	0.03 *	0.01	1.004 (1.001–1.008)
*Type of floor*		1		
Dirt	26 (54.2)			
Dirt and concrete	22 (45.8)			
*Main vegetation cover*				
Trees	21 (43.7)	0.48		
Schrubs	6 (12.5)	1		
Herbaceus	6 (12.5)	1		
*Dominant vegetation*		1		
Trees	28 (58.3)			
Herbaceous	20 (41.7)			
*Number of trees*	23.31 ± 14.87	0.48		
*Diversity of trees*	8.10 ± 3.74	0.08		
*Buildings in the peridomicile*				
Warehouse	21 (43.7)	0.48		
Barn	10 (20.8)	1		
Buildings for animal husbandry	23 (47.9)	0.29 *		
*Production animals*		0.46		
Absence	24 (50)			
One species	13 (27.1)			
More than one species	11 (22.9)			
*Captive wildlife*		0.3 *		
Absence	26 (54.2)			
One species	18 (37.5)			
More than one species	4 (8.4)			
*Accumulation of miscellaneous items*				
PET	35 (72.9)	0.1 *		
Cans	34 (70.8)	1		
Cardboard	9 (18.8)	1		
Pots	43 (89.5)	1		
Firewood	39 (81.2)	0.37		
Stones	34 (70.8)	0.45		
Construction materials	32 (66.7)	1		
Cement blocks	24 (50)	0.72		
Wood	4 (8.3)	1		
Construction clay	10 (20.8)	0.66		
Construction rubbish	6 (12.5)	1		
Gravel	4 (8.3)	0.56		
*Water containers*	45 (93.7)	0.51		

The variables marked with * were considered for the multivariate analysis. In the multivariate analysis, only the results of the variables analyzed with the selected model are reported for risk ratios (RR) and 95% confidence intervals (CI 95%).

**Table 5:** Deviance analysis comparing models to predict the probability of finding at least one seropositive animal against *Leptospira* according to the characteristics of the studied peridomiciles.

Models	AIC	Residual Deviance	df	Deviance	*p*
Model 1. Null model	44.47	49.13			
Model 2. Peridomicile area	52.61	40.47	1	8.65	0.003
Model 3. Number of trees	49.34	48.61	0	−8.14	
Model 4. Tree diversity	46.17	45.34	0	3.27	
Model 5. Area + tree diversity	51.13	40.2	1	5.17	0.02
Model 6. Area + geographic polygon	41.62	31.62	2	8.55	0.01

**Table 6:** Bivariate analysis (Fisher’s exact test) and the multinomial generalized linear model (GLM) fitted with the characteristics of the peridomiciles associated with one or more seropositive species per peridomicile (N = 48).

Peridomicile Characteristics	Number of Peridomiciles (%)	Bivariate Analysis (*p* ≤ 0.3)	Multinomial Analysis (*p* < 0.05)	RR (95%CI)
*Number of seropositive species per peridomicile*				
Zero	10 (20.8)			
One	25 (52)			
Two	13 (27)			
*Locality’s area*		0.007 *	Reference	
Interior	16 (33.3)		One species: 0.96	1.05 (0.14–7.55)
Central	16 (33.3)		Two species: 0.41	3.16 (0.19–51.41)
Exterior	16 (33.3)		One species: 0.10	0.17 (0.02–1.41)
			Two species: 0.14	6.62 (0.51–85.44)
*Geographic polygon*		0.61		
Northwest	9 (18.8)			
Northeast	10 (20.8)			
Southest	12 (25)			
Southwest	17 (35.4)			
*Type of neighboring*		0.59		
Houses	11 (22.9)			
Inhabited properties	23 (48)			
Public areas	14 (29.1)			
*Peridomicile delimiter*		0.30 *		
Block wall	4 (8.4)			
Stone wall	15 (31.2)			
Wall builded with diverse materials	15 (31.2)			
Without delimiter	14 (29.2)			
*Type of floor*		0.28 *		
Dirt	26 (54.2)			
Dirt and concrete	22 (45.8)			
*Main vegetation cover*				
Trees	21 (43.7)	0.24 *		
Schrubs	6 (12.5)	0.88		
Herbaceus	6 (12.5)	0.05 *		
*Dominant vegetation*		0.20 *		
Trees	28 (58.3)			
Herbaceous	20 (41.7)			
*Buildings in the peridomicile*				
Warehouse	21 (43.7)	0.24 *		
Barn	10 (20.8)	0.58		
Buildings for animal husbandry	23 (47.9)	0.40		
*Production animals*		0.71		
Absence	24 (50)			
One species	13 (27.1)			
More than one species	11 (22.9)			
*Captive wildlife*		0.47		
Absence	26 (54.2)			
One species	18 (37.5)			
More than one species	4 (8.4)			
*Accumulation of miscellaneous items*				
PET	35 (72.9)	0.03 *	One species: 0.02 *	8.52 (1.38–52.56)
Cans	34 (70.8)	0.005 *		
Cardboard	9 (18.8)	0.54	Two species: 0.67	1.46 (0.25–8.52)
Pots	43 (89.5)	0.15 *		
Firewood	39 (81.2)	0.62		
Stones	34 (70.8)	0.76		
Construction materials	32 (66.7)	0.92		
Cement blocks	24 (50)	0.86		
Wood	4 (8.3)	0.41		
Construction clay	10 (20.8)	0.46		
Construction rubbish	6 (12.5)	1		
Gravel	4 (8.3)	0.80		
*Water containers*	45 (93.7)	0.77		

Variables marked with * were considered for the multivariate analysis. In the multinomial GLM, only the results of the selected model are reported. RR= Risk Ratio.

## Data Availability

The raw data supporting the conclusions of this article will be made available by the authors upon request.

## References

[1] Ko A.I., Goarant C., Picardeau M. (2009). *Leptospira*: The dawn of the molecular genetics era for an emerging zoonotic pathogen. Nat. Rev. Microbiol..

[2] Mwachui M.A., Crump L., Hartskeerl R., Zinsstag J., Hattendorf J. (2015). Environmental and behavioural determinants of Leptospirosis transmission: A Systematic review. PLoS Negl. Trop. Dis..

[3] Casanovas-Massana A., Costa F., Riediger I.N., Cunha M., de Oliveira D., Mota D.C., Sousa E., Querino V.A., Nery N., Reis M.G. (2018). Spatial and temporal dynamics of pathogenic *Leptospira* in surface waters from the urban slum environment. Water Res..

[4] Casanovas-Massana A., Hamond C., Santos L.A., de Oliveira D., Hacker K.P., Balassiano I., Costa F., Medeiros M.A., Reis M.G., Ko A.I. (2020). *Leptospira yasudae* sp. nov. and *Leptospira stimsonii* sp. nov., two new species of the pathogenic group isolated from environmental sources. Int. J. Syst. Evol. Microbiol..

[5] Fernandes L.G.V., Stone N.E., Roe C.C., Goris M.G.A., van der Linden H., Sahl J.W., Wagner D.M., Nally J.E. (2022). *Leptospira sanjuanensis* sp. nov., a pathogenic species of the genus *Leptospira* isolated from soil in Puerto Rico. Int. J. Syst. Evol. Microbiol..

[6] Caimi K., Ruybal P. (2020). *Leptospira* spp., a genus in the stage of diversity and genomic data expansion. Infect. Genet. Evol..

[7] Costa F., Hagan J.E., Calcagno J., Kane M., Torgerson P., Martinez-Silveira M.S., Stein C., Abela-Ridder B., Ko A.I. (2015). Global morbidity and mortality of Leptospirosis: A systematic review. PLoS Negl. Trop. Dis..

[8] Browne E.S., Pereira M., Barreto A., Zeppelini C.G., de Oliveira D., Costa F. (2023). Prevalence of human leptospirosis in the Americas: A systematic review and meta-analysis. Rev. Panam. Salud Publica.

[9] Yescas-Benítez J.E., Rivero-Perez N., Monntiel-Díaz H.E., Valladares-Carranza B., Peláez-Acero A., Morales-Ubaldo A.L., Zaragoza-Bastida A. (2020). Comportamiento epidemiológico de la leptospirosis en México durante el periodo 2013–2010. Rev. Salud Pública.

[10] Boletín Epidemiológico Sistema Nacional de Vigilancia Epidemiológica Sistema Único de Información 2023. Secretaría de Salud. Dirección General de Epidemiología. https://www.gob.mx/salud/documentos/boletinepidemiologico-sistema-nacional-de-vigilancia-epidemiologica-sistema-unico-de-informacion-261547.

[11] Vado-Solis I., Cárdenas-Marrufo M.F., Jiménez-Delgadillo B., Alzina-López A., Laviada-Molina H., Suarez-Solís V., Zavala-Velázquez J.E. (2002). Clinical-epidemiological study of leptospirosis in humans and reservoirs in Yucatán, México. Rev. Inst. Med. Trop. Sao Paulo.

[12] Torres-Castro M.A., Gutiérrez-Ruíz E., Hernández-Betancourt S., Peláez-Sánchez R., Agudelo-Flórez P., Guillermo-Cordero L., Puerto F.I. (2014). First molecular evidence of *Leptospira* spp. in synanthropic rodents captured in Yucatan, Mexico. Rev. Méd Vét.

[13] Torres-Castro M., Cruz-Camargo B., Medina-Pinto R., Reyes-Hernández B., Moguel-Lehmer C., Medina R., Ortiz-Esquivel J., Arcila-Fuentes W., López-Ávila A., Noh-Pech H. (2018). Detección molecular de leptospiras patógenas en roedores sinantrópicos y silvestres capturados en Yucatán, México. Biomedica.

[14] Panti-May J.A., DE Andrade R.R.C., Gurubel-González Y., Palomo-Arjona E., Sodá-Tamayo L., Meza-Sulú J., Ramírez-Sierra M., Dumonteil E., Vidal-Martínez V.M., Machaín-Williams C. (2017). A survey of zoonotic pathogens carried by house mouse and black rat populations in Yucatan, Mexico. Epidemiol. Infect..

[15] Suárez-Galaz A., Reyes-Novelo E., Hernández-Betancourt S., Panti-May A., Estrella E., Sánchez-Montes S., Noh-Pech H., Lugo-Caballero C., Colunga-Salas P., Peláez-Sánchez R. (2024). Study on the relation of the characteristics of the capture sites with the *Leptospira* spp. occurrence in bats and rodents from Yucatan, Mexico. Acta Trop..

[16] Torres-Castro M., Febles-Solís V., Hernández-Betancourt S., Noh-Pech H., Estrella E., Peláez-Sánchez R., Panti-May A., Herrera-Flores B., Reyes-Hernández B., Sosa-Escalante J. (2020). *Leptospira* patógenas en murciélagos de Campeche y Yucatán, México. Rev. MVZ Córdoba.

[17] Torres-Castro M., Panti-May J.A., MacSwiney-González M.C., Lugo-Caballero C., Suárez-Galaz A., Suárez-Galaz M., Yeh-Gorocica A., Cruz-Camargo B. (2023). Detección de *Leptospira* spp. en murciélagos de la península de Yucatán, México. Rev. Cient. Fac. Vet..

[18] Ruiz-Piña H.A., Puc-Franco M.A., Flores-Abuxapqui J., Vado-Solis I., Cardenas-Marrufo M.F. (2002). Isolation of *Salmonella enterica* and serologic reactivity to *Leptospira interrogans* in opossums (*Didelphis virginiana*) from Yucatán, México. Rev. Inst. Med. Trop. Sao Paulo.

[19] Suárez-Galaz A.R., Hernández-Betancourt S., Panti-May J.A., Manrique-Saide P., Torres-Castro M. (2021). Evidencia de *Leptospira* spp. en musarañas *Cryptotis mayensis*. Nuevo hospedero en Yucatán, México. Rev. Biomedica.

[20] Jimenez-Coello M., Vado-Solis I., Cárdenas-Marrufo M.F., Rodríguez-Buenfil J.C., Ortega-Pacheco A. (2008). Serological survey of canine leptospirosis in the tropics of Yucatan Mexico using two different tests. Acta Trop..

[21] Ortega-Pacheco A., Guzmán-Marín E., Acosta-Viana K.Y., Vado-Solís I., Jiménez-Delgadillo B., Cárdenas-Marrufo M., Pérez-Osorio C., Puerto-Solís M., Jiménez-Coello M. (2017). Serological survey of *Leptospira interrogans*, *Toxoplasma gondii* and *Trypanosoma cruzi* in free roaming domestic dogs and cats from a marginated rural area of Yucatan Mexico. Vet. Med. Sci..

[22] Torres-Castro M., Díaz-Aceves D., Suárez-Galaz A., Reyes-Novelo E., Rodríguez-Vivas R.I. (2021). Evidencia de *Leptospira* spp. en sangre de perros de una comunidad rural de Yucatán, México. Rev. MVZ Córdoba.

[23] Zavala-Velázquez J., Pinzón-Cantarell J., Flores-Castillo M. (1984). La leptospirosis en Yucatán. Estudio serológico en humanos y animales. Salud Publica México.

[24] Agudelo-Flórez P., Restrepo-Jaramillo B.N., Arboleda-Naranjo M. (2007). Situación de la leptospirosis en el Urabá antioqueño colombiano: Estudio seroepidemiológico y factores de riesgo en población general urbana. Cad. Saude Publica.

[25] Reis R.B., Ribeiro G.S., Felzemburgh R.D., Santana F.S., Mohr S., Melendez A.X., Queiroz A., Santos A.C., Ravines R.R., Tassinari W.S. (2008). Impact of environment and social gradient on *Leptospira* infection in urban slums. PLoS Negl. Trop. Dis..

[26] Espinoza-Gómez F., Maldonado-Rodríguez A., Coll-Cardenas R., Hernandez-Suárez C.M., Fernández-Salas I. (2002). Presence of triatominae (Hemiptera, Reduviidae) and risk of transmission of Chagas disease in Colima, Mexico. Mem. Inst. Oswaldo Cruz.

[27] Reyes-Novelo E., Ruiz-Piña H., Canché-Pool E.B., Panti-May J.A., Escobedo-Ortegón F.J. (2022). El peridomicilio y las zoonosis en Yucatán. Hacia la búsqueda de Una Salud. Trop. Subtrop. Agroecosyst..

[28] Dzib-Paredes G., Rodríguez-Vivas R.I., Panti-May A., Noh-Pech H., Rosado-Aguilar J.A., Torres-Castro M. (2022). Frecuencia de *Borrelia burgdorferi* sensu lato y *Leptospira* spp. en pequeños roedores de Yucatán, México. Rev. Cient. Fac. Vet..

[29] Ruiz-Piña H.A., Reyes-Novelo E.A., Salvador-Flores J. (2012). El huerto familiar yucateco y las zoonosis. Huertos Familiares de la Península de Yucatán.

[30] Instituto Nacional de Estadística y Geografía Compendio de Información Geográfica Municipal 2010. Ucú. Yucatán. https://www.inegi.org.mx/contenidos/app/mexicocifras/datos_geograficos/31/31100.pdf#page=2.09.

[31] Instituto Nacional de Estadística y Geografía Espacio y Datos de México. https://www.inegi.org.mx/app/mapa/espacioydatos/default.aspx?ag=311000001.

[32] Panti-May J.A., Hernández-Betancourt S., Ruíz-Piña H., Medina-Peralta S. (2012). Abundance and population parameters of commensal rodents present in rural households in Yucatan, Mexico. Int. Biodeterior. Biodegrad..

[33] Panti-May J.A., Hernández-Betancourt S.F., Torres-Castro M.A., Parada-López J., López-Manzanero S., Herrera-Meza M. (2018). A population study of the house mouse, *Mus. musculus* (Rodentia: Muridae), in a rural community of Mérida, México. Caribb. Nat..

[34] Thrusfield M. (2018). Veterinary Epidemiology. Presenting Numerical Data.

[35] Ponce-Saavedra J., Quijano-Ravell A.F., Valdez-Mondragón A., Zuria I., Olvera-Ramírez A.M., Ramírez-Bastida P. (2019). Técnicas para la recolección de arañas y otros arácnidos en ambientes antrópicos. Manual de Técnicas Para el Estudio de Fauna Nativa en Ambientes Urbanos.

[36] Hernández-Camacho N., Muñoz-García C.I., Ruiz-Piña H.A., Reyes-Novelo E.A., Olvera-Ramírez A.M., Zuria I., Olvera-Ramírez A.M., Ramírez-Bastida P. (2019). Técnicas de manejo de hospederos y colecta de parásitos de vertebrados urbanos. Manual de Técnicas Para el Estudio de Fauna Nativa en Ambientes Urbanos.

[37] AVMA Guidelines for the Euthanasia of Animals: 2020 Edition*. https://www.avma.org/sites/default/files/2020-02/Guidelines-on-Euthanasia-2020.pdf.

[38] Barrera-Tolosa M.S. (2020). Elaboración de un Manual de Rehabilitación Para la Especie Didelphis Marsupialis Alojados en el CAV-CEARFS de la CDMB. Bachelor’s Thesis.

[39] World Organisation for Animal Health Manual of Diagnosis Tests and Vaccines for Terrestrial Animals, Thirteenth Edition 2024. Chapter 3.1.12. Leptospirosis. https://www.woah.org/fileadmin/Home/eng/Health_standards/tahm/3.01.12_LEPTO.pdf.

[40] Donald D.M. (1983). Manual de Métodos Para el Diagnóstico de Laboratorio de la Leptospirosis.

[41] Carrillo-Peraza J.R., Manrique-Saide P., Rodríguez-Buenfil J.C., Escobedo-Ortegón J.F., Rodríguez-Vivas R.I., Bolio-González M.E., Barrera-Pérez M., Reyes-Novelo E., Sauri-Arceo C.H. (2014). Estudio serológico de la tripanosomiasis americana y factores asociados en perros de una comunidad rural de Yucatán, México. Arch. Med. Vet..

[42] Koyoc-Cardeña E., Medina-Barreiro A., Escobedo-Ortegón F.J., Rodríguez-Buenfil J.C., Barrera-Pérez M., Reyes-Novelo E., Chablé-Santos J., Selem-Salas C., Vazquez-Prokopec G., Manrique-Saide P. (2015). Chicken coops, *Triatoma dimidiata* infestation and its infection with *Trypanosoma cruzi* in a rural village of Yucatán, Mexico. Rev. Inst. Med. Trop. Sao Paulo.

[43] Torres-Castro M., Reyes-Novelo E., Noh-Pech H., Tello-Martín R., Lugo-Caballero C., Dzul-Rosado K., Puerto-Manzano F., Rodríguez-Vivas R.I. (2020). Personal and household factors involved in recent *Rickettsia* exposure in a rural population from Yucatán, Mexico. Zoonoses Public Health.

[44] Dzul-Rosado K.R., Reyes-Novelo E., Lugo-Caballero C., Cuxim-Koyoc A.D., Collí-Padrón F., Tello-Martín R., López-Ávila K.B., Palma-Chan A., Peniche-Lara G., Ruiz-Piña H.A. (2021). Urban ecology of hosts and vectors of *Rickettsia* in a rickettsiosis-endemic city of the Yucatan peninsula, Mexico. Acta Trop..

[45] Reiczigel J., Marozzi M., Fábián I., Rózsa L. (2019). Biostatistics for parasitologists—A primer to Quantitative Parasitology. Trends Parasitol..

[46] Posit Team RStudio: Integrated Development Environment for R. Posit Software.

[47] R Core Team (2013). R: A Language and Environment for Statistical Computing.

[48] Fox J., Weisberg S. (2019). An R Companion to Applied Regression.

[49] Ferreira E.B., Cavalcanti P.P., Nogueira D.A. ExpDes: Pacote Experimental Designs.

[50] Gross J., Ligges U. Tests for Normality. Package ‘nortest’. Version 1.0-4. https://cran.r-project.org/web/packages/nortest/nortest.pdf.

[51] Russell L. Least-Squares Means. Package ‘lsmeans’. Version 2.30-0. https://cran.r-project.org/web/packages/lsmeans/lsmeans.pdf.

[52] Lenth R.V., Bolker B., Buerkner P., Giné-Vázquez I., Herve M., Jung M., Love J., Miguez F., Piaskowski J., Riebl H. Estimated Marginal Means, aka Least-Squares Means. Package ‘emmeans’. Version 1.10.4. https://cran.r-project.org/web/packages/emmeans/emmeans.pdf.

[53] De Rosario-Martínez H., Fox J., Phil C., R Core Team Post-Hoc Interaction Analysis. Package ‘Phia’. Version 0.3-1. https://cran.r-project.org/web/packages/phia/phia.pdf.

[54] Wickham H., Chang W., Henry L., Pedersen T.L., Takahashi K., Wilke C., Woo K., Yutani H., Dunnington D., van den Brand T. PBC, Posit. ggplot2: Create Elegant Data Visualisations Using the Grammar of Graphics. Version 3.5.1. https://cran.r-project.org/web/packages/ggplot2/index.html.

[55] Ripley B., Venables W. Feed-Forward Neural Networks and Multinomial Log-Linear Models. Package ‘nnet’. Version 7.3-19. https://cran.r-project.org/web/packages/nnet/nnet.pdf.

[56] Wickham H. reshape2: Flexibly Reshape Data: A Reboot of the Reshape Package. Package ‘reshape2’. Version 1.4.4. https://cran.r-project.org/web/packages/reshape2/reshape2.pdf.

[57] Pinheiro J., Bates D., DebRoy S., Sarkar D., Heisterkamp S., Van Willigen B., Ranke J., EISPACK Authors, R Core Team Linear and Nonlinear Mixed Effects Models. Package ‘nlme’. Version 3.1-166. https://cran.r-project.org/web/packages/nlme/nlme.pdf.

[58] Ripley B., Venables B., Bates D.M., Hornik K., Gebhardt A., Firth D. Support Functions and Datasets for Venables and Ripley’s MASS. Version 7.3-61. https://cran.r-project.org/web/packages/MASS/MASS.pdf.

[59] Fox J., Marquez M., Muenchen R., Putler D. R Commander Miscellaneous Functions. Package ‘RcmdrMisc’. Version 2.9-1. https://cran.r-project.org/web/packages/RcmdrMisc/RcmdrMisc.pdf.

[60] Garcia-Lopez M., Lurier T., Bouilloud M., Pradel J., Tatard C., Sepulveda D., Anfray G., Dussert J., Bourhy P., Charbonnel N. (2024). Prevalence, genetic diversity and eco-epidemiology of pathogenic *Leptospira* species in small mammal communities in urban parks Lyon city, France. PLoS ONE.

[61] Adler B., de la Peña-Moctezuma A. (2010). *Leptospira* and leptospirosis. Vet. Microbiol..

[62] Méndez C., Benavides L., Esquivel A., Aldama A., Torres J., Gavaldón D., Meléndez P., Moles L. (2013). Pesquisa serológica de *Leptospira* en roedores silvestres, bovinos, equinos y caninos en el noreste de México. Rev. Salud Anim..

[63] Levett P.N. (2001). Leptospirosis. Clin. Microbiol. Rev..

[64] Fornazari F., Langoni H., Marson P.M., Nóbrega D.B., Teixeira C.R. (2018). *Leptospira* reservoirs among wildlife in Brazil: Beyond rodents. Acta Trop..

[65] Horta M.C., Ragozo A.M.A., Casagrande R.A., Reiko E., Gennari S.M. (2016). Occurrence of anti-*Toxoplasma gondii*, *Neospora caninum* and *Leptospira* spp. antibodies in opossums (*Didelphis* spp.) in São. Braz. J. Vet. Res. Anim. Sci..

[66] Jorge S., Hartleben C.P., Seixas F.K., Coimbra M.A., Stark C.B., Larrondo A.G., Amaral M.G., Albano A.P., Minello L.F., Dellagostin O.A. (2012). *Leptospira borgpetersenii* from free-living white-eared opossum (*Didelphis albiventris*): First isolation in Brazil. Acta Trop..

[67] Medeiros L.D.S., Braga-Domingos S.C., Azevedo M.I.N.D., Peruquetti R.C., de Albuquerque N.F., D’Andrea P.S., Botelho A.L.M., Crisóstomo C.F., Vieira A.S., Martins G. (2020). Small mammals as carriers/hosts of *Leptospira* spp. in the western Amazon forest. Front. Vet. Sci..

[68] Fernandes J.J., de Lima-Peixoto A., de Farias A.S.S., Junior-Pinheiro T., da Costa D.F., Silva M.L.C.R., Júnior J.P.A., Malossi C.D., Ullmann L.S., de Azevedo S.S. (2020). *Didelphis albiventris* as a carrier of *Leptospira* sp. in the central nervous tissue in the semiarid region of Northeast, Brazil. Comp. Immunol. Microbiol. Infect. Dis..

[69] Vieira A.S., D’Andrea P.S., Vilela R.D.V., Loretto D., Jaeger L.H., Carvalho-Costa F.A., Lilenbaum W. (2019). Pathogenic *Leptospira* species are widely disseminated among small mammals in Atlantic Forest biome. Transbound. Emerg. Dis..

[70] Ruiz-Piña H., Pacheco-Castro J., Lugo-Pérez J.A., Pacheco-Castro J., Lugo-Pérez J.A., Tzuc-Canché L., Ruíz-Piña H.A. (2013). El “zorro” de Yucatán y su relación con la población humana. Estudios Multidisciplinarios de Las Enfermedades Zoonóticas y ETVs en Yucatán.

[71] Ortega-Pacheco A., Colin-Flores R.F., Gutiérrez-Blanco E., Jiménez-Coello M. (2008). Frequency and type of renal lesions in dogs naturally infected with *Leptospira* species. Ann. N. Y. Acad. Sci..

[72] Cardenas-Marrufo M.F., Vado-Solis I., Perez-Osorio C.E., Correa J.S. (2011). Seropositivity to leptospirosis in domestic reservoirs and detection of *Leptospira* spp. from water sources, in farms of Yucatan, Mexico. Trop. Subtrop. Agroecosyst..

[73] Blum-Domínguez S.d.C., Chi-Dzib M.Y., Maldonado-Velázquez M.G., Nuñez-Oreza L.A., Gómez-Solano M.I., Caballero-Poot R.I., Tamay-Segovia P. (2013). Detection of reactive canines to *Leptospira* in Campeche City, Mexico. Rev. Argent Microbiol..

[74] Cruz-Romero A., Gil-Alarcón G., Ochoa-Valencia J.L., Ramos-Vásquez J.R., Romero-Salas D., Becker I., Sánchez-Montes S., Arenas P. (2024). Seroprevalencia de Leptospira en perros ferales de la Reserva Ecológica del Pedregal de San Ángel, México. Rev. Cient. Fac. Vet..

[75] Jimenez-Coello M., Ortega-Pacheco A., Guzman-Marin E., Guiris-Andrade D.M., Martinez-Figueroa L., Acosta-Viana K.Y. (2010). Stray dogs as reservoirs of the zoonotic agents *Leptospira interrogans*, *Trypanosoma cruzi*, and *Aspergillus* spp. in an urban area of Chiapas in southern Mexico. Vector Borne Zoonotic Dis..

[76] Goldstein R.E. (2010). Canine leptospirosis. Vet. Clin. North Am. Small Anim. Pract..

[77] Schuller S., Francey T., Hartmann K., Hugonnard M., Kohn B., Nally J.E., Sykes J. (2015). European consensus statement on leptospirosis in dogs and cats. J. Small Anim. Pract..

[78] Balboni A., Mazzotta E., Boniotti M.B., Bertasio C., Bellinati L., Lucchese L., Battilani M., Ceglie L., Marchione S., Esposito G. (2022). Outbreak of *Leptospira borgpetersenii* serogroup Sejroe infection in kennel: The role of dogs as sentinel in specific environments. Int. J. Environ. Res. Public Health.

[79] Ellis W.A. (2015). Animal leptospirosis. Curr. Top. Microbiol. Immunol..

[80] Sykes J.E., Francey T., Schuller S., Stoddard R.A., Cowgill L.D., Moore G.E. (2023). Updated ACVIM consensus statement on leptospirosis in dogs. J. Vet. Intern. Med..

[81] Zamri M.I.M., Shafie N.J., Ali M.R.M., Awoniyi A.M., Argibay H.D., Costa F. (2024). Socio-environmental factors associated with small mammal assemblage and *Leptospira* prevalence in Suburban Areas of Terengganu, Malaysia. Asian Pac. J. Trop. Med..

[82] Salazar-Barrientos L.d.L., Magaña-Magaña M.A., Latourneirie-Moreno L. (2015). Importancia económica y social de la agrodiversidad del traspatio en una comunidad rural de Yucatán, México. Agric. Soc. Desarro..

[83] Ruiz-Piña H.A., Reyes-Novelo E., Escobedo-Ortegón F.J., Barrera-Pérez M.A., Salvador-Flores J. (2012). Mamíferos sinantrópicos y la transmisión de enfermedades zoonóticas en el área rural de Yucatán. Huertos Familiares de la Península de Yucatán.

[84] Herrera-Flores B.G., Santos-Fita D., Naranjo E.J., Hernández-Betancourt S. (2019). Importancia cultural de la fauna silvestre en comunidades rurales del norte de Yucatán, México. Peninsula.

[85] Nahuat-Cervera P.E., Estrada-Riaño I.A., Peraza-Romero F., Uitzil-Collí M.O., Basora-Dorantes R.A., de los Á. Basora-Dorantes S. (2021). Conocimiento y aprovechamiento tradicional de vertebrados silvestres en la comunidad maya de Zavala, municipio de Sotuta, Yucatán, México. Estud. Cult. Maya.

[86] Richardson D.J., Gauthier J.L. (2003). A serosurvey of leptospirosis in Connecticut peridomestic wildlife. Vector Borne Zoonotic Dis..

[87] Helman S.K., Tokuyama A.F.N., Mummah R.O., Stone N.E., Gamble M.W., Snedden C.E., Borremans B., Gomez A.C.R., Cox C., Nussbaum J. (2023). Pathogenic *Leptospira* are widespread in the urban wildlife of southern California. Sci. Rep..

[88] Della Rossa P., Tantrakarnapa K., Sutdan D., Kasetsinsombat K., Cosson J.F., Supputamongkol Y., Chaisiri K., Tran A., Supputamongkol S., Binot A. (2016). Environmental factors and public health policy associated with human and rodent infection by leptospirosis: A land cover-based study in Nan province, Thailand. Epidemiol. Infect..

[89] McMahon B.J., Morand S., Gray J.S. (2018). Ecosystem change and zoonoses in the Anthropocene. Zoonoses Public Health.

[90] Richard E., Bourhy P., Picardeau M., Moulin L., Wurtzer S. (2021). Effect of disinfection agents and quantification of potentially viable *Leptospira* in freshwater samples using a highly sensitive integrity-qPCR assay. PLoS ONE.

[91] Canul-Bacab F., May-Hoil P.E. (2016). El problema de la basura en el interior del estado de Yucatán. Reaxion.

[92] Pacheco-Castro J., Lugo-Pérez J.A., Tzuc-Canché L.M., Salvador-Flores J. (2012). Relación de variables socioeconómicas y culturales con la prevalencia de enfermedades zoonóticas y ETVs en Molas. Huertos Familiares de la Península de Yucatán.

